# Synergistic study of a Danshen (Salvia Miltiorrhizae Radix et Rhizoma) and Sanqi (Notoginseng Radix et Rhizoma) combination on cell survival in EA.hy926 cells

**DOI:** 10.1186/s12906-019-2458-z

**Published:** 2019-02-21

**Authors:** Xian Zhou, Valentina Razmovski-Naumovski, Antony Kam, Dennis Chang, Chun Guang Li, Kelvin Chan, Alan Bensoussan

**Affiliations:** 1 NICM Health Research Institute, Western Sydney University, Sydney, 2751 Australia; 2 South Western Sydney Clinical School, UNSW Medicine, University of New South Wales Sydney, Sydney, 2170 Australia; 3 School of Biological Sciences, Nanyang Technological University, Singapore, 637551 Singapore; 4 School of Pharmacy and Biomolecular Sciences, Liverpool John Moores University, L3 3AF, Liverpool, UK

**Keywords:** Danshen-Sanqi, Synergy, Cell injury, Oxidative stress, Homocysteine, Tumour necrosis factor, Combination index

## Abstract

**Background:**

This study investigated the protective effects of the *Danshen* (DS) and *Sanqi* (SQ) herb pair on cell survival in the human cardiovascular endothelial (EA.hy926) cell line exposed to injury.

**Methods:**

Nine combination ratios of Danshen-Sanqi extracts (DS-SQ) were screened for their protective effects in the EA.hy926 cell line against two different cellular impairments induced by DL-homocysteine (Hcy) – adenosine (Ado) – tumour necrosis factors (TNF) and oxidative stress (H_2_O_2_), respectively. The type of interaction (synergistic, antagonistic, additive) between DS and SQ was analysed using a combination index (CI) model. The effects of key bioactive compounds from DS and SQ were tested using the same models. The compound from each herb that demonstrated the most potent activity in cell viability was combined to evaluate their synergistic/antagonistic interaction using CI.

**Results:**

DS-SQ ratios of 6:4 (50–300 μg/mL) produced synergistic effects (CI < 1) in restoring cell viability, reducing lactate dehydrogenase (LDH) leakage and caspase-3 expressions against Hcy-Ado-TNF. Additionally, DS-SQ 6:4 (50–150 μg/mL) was found to synergistically protect endothelial cells from impaired cellular injury induced by oxidative damage (H_2_O_2_) by restoring reduced cell viability and inhibiting excessive expression of reactive oxygen species (ROS). In particular, the combination of salvianolic acid A (SA) and ginsenoside Rb1 (Rb1) at 4:6 (1–150 μM) showed synergistic effects in preventing cytotoxic effects caused by Hcy-Ado-TNF (CI < 1). This simplified combination also demonstrated synergistic effects on H_2_O_2_-induced oxidative damage on EA.hy926 cells.

**Conclusions:**

This study provides scientific evidence to support the traditional use of the DS-SQ combination on protecting endothelial cells through their synergistic interactions.

**Electronic supplementary material:**

The online version of this article (10.1186/s12906-019-2458-z) contains supplementary material, which is available to authorized users.

## Background

It is well known that complex pathological conditions require combinational therapies that can act on multiple biological targets to efficiently manage and treat the underlying mechanistic pathways. In modern medical research, synergy can be understood as augmented bioactivity of compounds on the same target/receptor, and/or multi-target behaviour, and/or enhanced bioavailability. This produces an effect which is greater than the sum of the effect from the individual agents [1]. Although modern medicine has recently developed multiple active drugs based on this synergy concept, traditional Chinese medicine (TCM) has incorporated synergy through herbal prescriptions for centuries. It is believed that multiple ingredients in a herbal formula could enhance the therapeutic outcome, reduce toxicity and systematically manage the complexities of the condition [1].

Endothelial dysfunction is an early marker of vascular dysfunction prior to the development of vascular structural changes and clinical symptoms. This contributes to the progression of atherosclerotic plaques and leads to various types of vascular diseases [2–4]. There are many risk factors that are related to endothelial dysfunction. For example, it has been repeatedly demonstrated that an elevated level of homocysteine (Hcy) in blood is an independent risk factor for atherosclerotic vascular disease affecting the coronary, cerebral and peripheral arteries [5–8]. Coupled with adenosine (Ado), S-adenosylhomocysteine accumulates and leads to cellular DNA hypomethylation [9, 10], which disrupts cell survival and results in cellular injury [10]. Previous literature has reported that tumour necrosis factor (TNF) not only has a direct impact on endothelial dysfunction (by down-regulating endothelial nitric oxide synthase (eNOS) expression), but is also associated with endothelial cell apoptosis by modulating the interactions of cell apoptosis suppressors and inducers [11, 12]. Several in vitro studies reported that Hcy and TNF with Ado significantly impaired endothelial cell survival and induced cell apoptosis [3, 13].

Reactive oxygen species (ROS) is another important biomarker for detecting endothelial cell death in endothelial dysfunction. It is known to induce endothelial cell death by modulating a series of intracellular signaling pathways [14, 15]. ROS directly reacts with eNOS and forms peroxynitrite, which triggers endothelium dysfunction [16–18]. In in vitro studies, H_2_O_2_-induced endothelial apoptosis has been extensively used to induce cellular injury caused by oxidative stress [19]. Given the complexity of the pathological pathways of endothelial dysfunction, a combinational therapy that can multi-target those pathways may be considered as a better option than using a single agent only.

The herb-pair of Danshen-Sanqi (DS-SQ) has been widely used in Chinese herbal medicines in Asian countries for the prevention and treatment of vascular diseases, including angina pectoris, stroke and myocardial infarction [20]. A study by Zeng et al (2006) revealed that the combination of DS-SQ at 5:3 and 1:1 showed potent protective effects on human umbilical vein endothelial cells (HUVECs) against hypoxia [21]. There are numerous in vivo and in vitro studies that have demonstrated the protective effects of DS and SQ as a single extract on cell injury/apoptosis induced by various stimulants. Moreover, these studies have elucidated the multi-target activities attributed to its chemical compounds. For example, the aqueous extract of DS prevented oxysterol-induced endothelial cell apoptosis in Sprague-Dawley rats [22] and reduced the infarct volume size in cerebral ischaemia reperfusion (CIR) rats [23]. DS extract exhibited anti-apoptotic activity using platelet-derived growth factor (PDGF)-BB (20 ng/mL) and TNF (10 ng/mL) stimulated-HUVECs via mitogen-activated protein kinase (MAPK) and NF-κB signalling pathways [24]. Additionally, studies showed that the anti-apoptotic effects were attributed to phenolic acids including salvianolic acid A (SA) [25–28] and salvianolic acid B (SB) [29–32], and tanshinones such as tanshinone IIA (TIIA) and cryptotanshinone (CT) [33–35]. Although the effects of SQ and its chemical compounds have yet been investigated on endothelial cells, several studies have shown the protective effects of notoginseng saponins on bone marrow stromal cells (BMSCs) induced by oxidative stress [36]. Among all major bioactives in SQ, ginsenoside Rd was the most extensively investigated compound for its anti-apoptotic activity in various cell lines [37, 38]. Although there have been several studies on the protective actions of DS and SQ as individual herbs on various cell lines including endothelial cells, their combinational effects remain to be investigated.

Therefore, this study reports the combination effects of DS-SQ on protecting endothelial cells against endothelial injuries induced by Hcy-Ado-TNF and oxidative stress (H_2_O_2_). Combination index (CI), a popular mathematical model, is used in this study to investigate the interaction (synergism, addition or antagonism) of the DS-SQ combination [23]. In addition, the underlying mechanistic behaviour of the synergistic interactions were further investigated by the evaluating the associated activities of purified major bioactives of these herbs.

## Methods

### Cell line and culture conditions

Human cardiovascular endothelial cell line (EA.hy926) was provided by Dr. Shanhong Ling (Monash University Central Clinical School, Australia). The cell line was cultured in DMEM/Ham’s F12 containing 15 mM HEPES and L-glutamine and supplemented with 10% FBS, 100 U/mL of penicillin and streptomycin (Gibco BRL, Australia). The cell line was grown in a 5% CO_2_-humidified incubator at 37 °C. Human TNF recombinant protein, the cell culture reagents including Dulbecco’s Modified Eagle’s Medium (DMEM)/Ham’s F12 containing 15 mM HEPES and L-glutamine and foetal bovine serum were purchased from Life Technologies (Australia). Penstrep (penicillin and streptomycin) was purchased from Gibco™ (Australia). All chemicals and reagents, including Hcy and Ado, were from Sigma-Aldrich (Australia) unless otherwise stated.

### Preparation of herbal samples and their bioactive compounds

Crude DS and SQ herbal materials were sourced from PuraPharm International Ltd., Hong Kong. They were labeled with a reference number and kept in the herbal laboratory of NICM Health Research Institute, Australia. The raw herbal materials were authenticated by Professor Si-bao Chen from the Department of Applied Biology and Chemical Technology, the Hong Kong Polytechnic University, Hong Kong, China, according to the Hong Kong Materia Medica Standards. The aqueous extracts were prepared as follows: 1 g of ground DS/SQ crude herbal powder (30-meshsize) was weighed and soaked with 30 mL water for 0.5 h, followed by refluxing with boiling water for another 1 h. The solution was then centrifuged at 3000 rpm for 5 min and the supernatant was separated and evaporated to dryness using a freeze dryer. For the preparation of the combined aqueous extracts of DS-SQ, the crude herbal powder of DS and SQ was combined in nine different ratios (1:9, 2:8, 3:7,..., 8:2, 9:1, *w*/w) and was extracted in the same manner as for the single extract. The chemical fingerprint of DS and SQ are shown in Additional file 1. The content of chemical standards in both single and combined extracts of DS and SQ is shown in Additional file 2.

Chemical standards for DS including sodium danshensu (DSS), salvianolic acid B (SB), salvianolic acid A (SA), tanshinone TIIA (TIIA), dihydrotanshinone I (DT), tanshinone I (TI) and cryptotanshinone (CT); and those for SQ including ginsenoside Rg1 (Rg1), ginsenoside Rg2 (Rg2), ginsenoside Rd (Rd), ginsenoside Rb1 (Rb1) and notoginsenoside R1 (NR1) were purchased from Chengdu Biopurify Phytochemicals Ltd. (Chengdu, China; purity > 98%) and were verified via liquid chromatography-mass spectrometry. The most potent bioactives from each extract were further combined in nine different ratios (1:9, 2:8, 3:7,..., 8:2, 9:1, *v*/v) and were tested using the same models as for the extracts. The standard stock solutions of these reference compounds were prepared in methanol and stored at 4^∘^C until further use.

### Hcy-ado-TNF induced cell injury

EA.hy926 cells were seeded on a 96-well cell culture plate for 24–48 h until confluent. DS, SQ, DS-SQ extracts (10–150 μg/mL), and their bioactive compounds (0.1–150 μM) [25–28] were treated on EA.hy926 cells for 4 h, followed by Hcy (0.5 mM), Ado (0.5 mM) and TNF (0.5 ng/mL) for another 20 h before further analysis [3, 13].

### Hcy-ado-TNF induced cell injury determined by MTT assay

After the treatment period, MTT solution (final concentration of 0.5 mg/mL in PBS) was added to the cells and incubated for 4 h at 37 °C. Dimethyl sulfoxide (DMSO) was then added to dissolve the insoluble formazan crystal. The absorbance was measured at 540 nm using a microplate reader (BMG Labtech Fluostar Optima, Mount Eliza, Victoria, Australia) [3, 13]. The density of formazan formed in the control (medium with vehicle) cells was taken as 100% of cell viability.

### Hcy-ado-TNF induced cell injury determined by LDH leakage assay

After the treatment period, the supernatants from the cells were subjected to LDH measurement using a commercial kit according to the manufacturer’s instructions [39]. Briefly, the cells in the 96-well cell culture plate with supernatant after the treatment period were centrifuged at 250 x g for 4 min. Then, 50 μL of the supernatant was transferred from each tested well to a fresh 96-well flat-bottom plate. Prepared CytoTox 96 substrate mix (50 μL) obtained from the kit was added to each well of the new 96-well plate containing samples transferred from the original cytotoxicity assay plate. The plate was then covered with aluminum foil to avoid light and incubated for 30 min at room temperature. Finally, 50 μL of stop solution (from the kit) was added to each well and the absorbance was monitored at 490 nm.

### Hcy-ado-TNF induced cell injury determined by caspase-3 activity colorimetric assay

A quantitative caspase-3 activity assay was conducted according to instructions of the R&D assay kit manufacturer, with minor modifications [40]. EA.hy926 cells were seeded in six-well flat-bottom cell culture plates and incubated for 24–48 h until the cells were confluent. After the treatment period for 4 h, the cells were collected by centrifugation at 250 x g for 10 min. The supernatant was gently removed, and the cell pellet was collected and lysed by the lysis buffer provided in the kit. After incubating the cell lysate on ice for 10 min, the protein was collected by centrifugation at 10,000 X g for 1 min. The total protein content was quantified by a Pierce BCA Protein Assay Kit (Thermo Fisher Scientific, Australia), and then the cell lysate was diluted to an approximate total protein concentration of 1 mg/mL. The cell lysate (50 μL) was transferred to a fresh 96-well flat-bottom plate and was mixed with 50 μL reaction buffer (1% DTT, *v*/v) and 5 μL of caspase-3 colorimetric substrate (DEVE-pNA). The plate was incubated at 37 °C overnight, and the absorbance was read on a microplate reader (BMG Labtech Fluostar Optima, Mount Eliza, Victoria, Australia) at a wavelength of 410 nm.

### Oxidative stress (H_2_O_2_) induced cell injury

To investigate the protective effects of DS, SQ, DS-SQ extracts and their bioactive compounds (0.1–150 μM) on endothelial cell toxicity caused by hydrogen peroxide (H_2_O_2_) [25–28], EA.hy926 cells were pre-treated with increasing concentrations of the herbal extracts (10–300 μg/mL) for 30 min, followed by H_2_O_2_ (0.5 mM) incubation for 20 h.

### H_2_O_2_ induced cell injury determined by intracellular ROS production

The assay was conducted according to the instruction of the cellular ROS detection assay kit manufacturer (Abcam, Australia) [19]. EA.hy926 cells were seeded on a 96-well cell culture plate at a concentration of 2.5 × 10^5^ cells/mL and allowed to reach confluence overnight. The medium was then removed, and the cells were washed once with 1X assay buffer (from the kit). The cells were than stained with 2′,7′ –dichlorofluorescin diacetate (DCFDA) (20 μM) at 100 μL per well. The plate was incubated with a staining solution for 45 min at 37 °C in the dark. After the incubation, the DCFDA staining was discarded and the plate’s initial absorbance (A_0_) was immediately read under a microplate reader (BMG Labtech Fluostar Optima, Mount Eliza, Victoria, Australia). The cells were then washed again with PBS, and tested herbal extracts of DS, SQ, DS-SQ with increasing concentrations (10–150 μg/mL) were added to the cells and treated for 1 h. The absorbance at the endpoint (A_1_) was measured in the presence of the treatments under the same microplate reader. The wavelength was set up with excitation at 455 nm and emission 535 nm at fluorescence mode. The final absorbance was calculated as A_1_ normalised with its corresponding A_0_ (A_1_/A_0_).

### Synergism determination

CI models are practical methods for evaluating the interactions of multiple agents on the same target. Computational software, CompuSyn (Biosoft, US), was used to analyse the interaction between DS and SQ on each of the anti-apoptosis bioassays.

For the analysis, the concentration-response curves of DS, SQ and DS-SQ on the same bioassay were generated. The data of each concentration and response were entered into the CompuSyn software 2.0. The specific measurement for the CI value of a combination of two constituents was based on Chou-Talalay method, and the equation for the calculation of CI value is shown below [1, 41]:

$$ CI=\frac{d1}{D1}+\frac{d2}{D2} $$

In this equation, d1 and d2 refer to the concentrations of constituent 1 (DS) and 2 (SQ) used alone to reach certain effect level, whereas D1 and D2 refers to the dose of constituent 1 (DS) and 2 (SQ) in the combination 12 (DS-SQ) to reach the same effect level, respectively.

Through the software process, the CI-Fa (fraction affected level) curve and the relevant statistic regarding the synergistic/antagonistic interactions were then generated automatically [1]. In this model, the CI value demonstrates the interaction level, where CI < 1, CI = 1 and CI > 1 suggest synergistic, additive and antagonistic interaction, respectively. Here, the CI-Fa curve demonstrates the relationship between the CI value and the various effective level (Fa). For example, CI = 1 at ED_50_ suggests that an additive effect is found (CI = 1) when the effect reaches 50% (ED_50_). Thus, the synergistic/antagonistic interaction at effective ranges can be interpreted through this CI-Fa curve.

### Statistical analysis

All statistic comparisons were performed using GraphPad Version 5.02 (US). The significance was analysed by one-way ANOVA test. Data was expressed as a mean ± SEM, *n* > 3. *P* < 0.05 was considered statistically significant.

## Results

### Synergistic effects of DS, SQ and DS-SQ and their bioactive compounds on Hcy-ado-TNF impaired cell survival in EA.hy926 cells

#### Synergistic effects of DS, SQ and DS-SQ on impaired cell viability

The cytotoxic effects of TNF associated with Hcy and Ado were demonstrated in a concentration-dependent manner in EA.hy926 cells. A steadily decrease in cell viability was observed when Hcy and Ado were co-incubated with TNF (0.1–10 ng/mL) in EA.hy926 cells. Significant cell viability was reduced by over 40% in comparison to blank control when the concentration of TNF was greater than 0.5 ng/mL (*P* < 0.01) (Additional file 3).

**Fig. 1:**
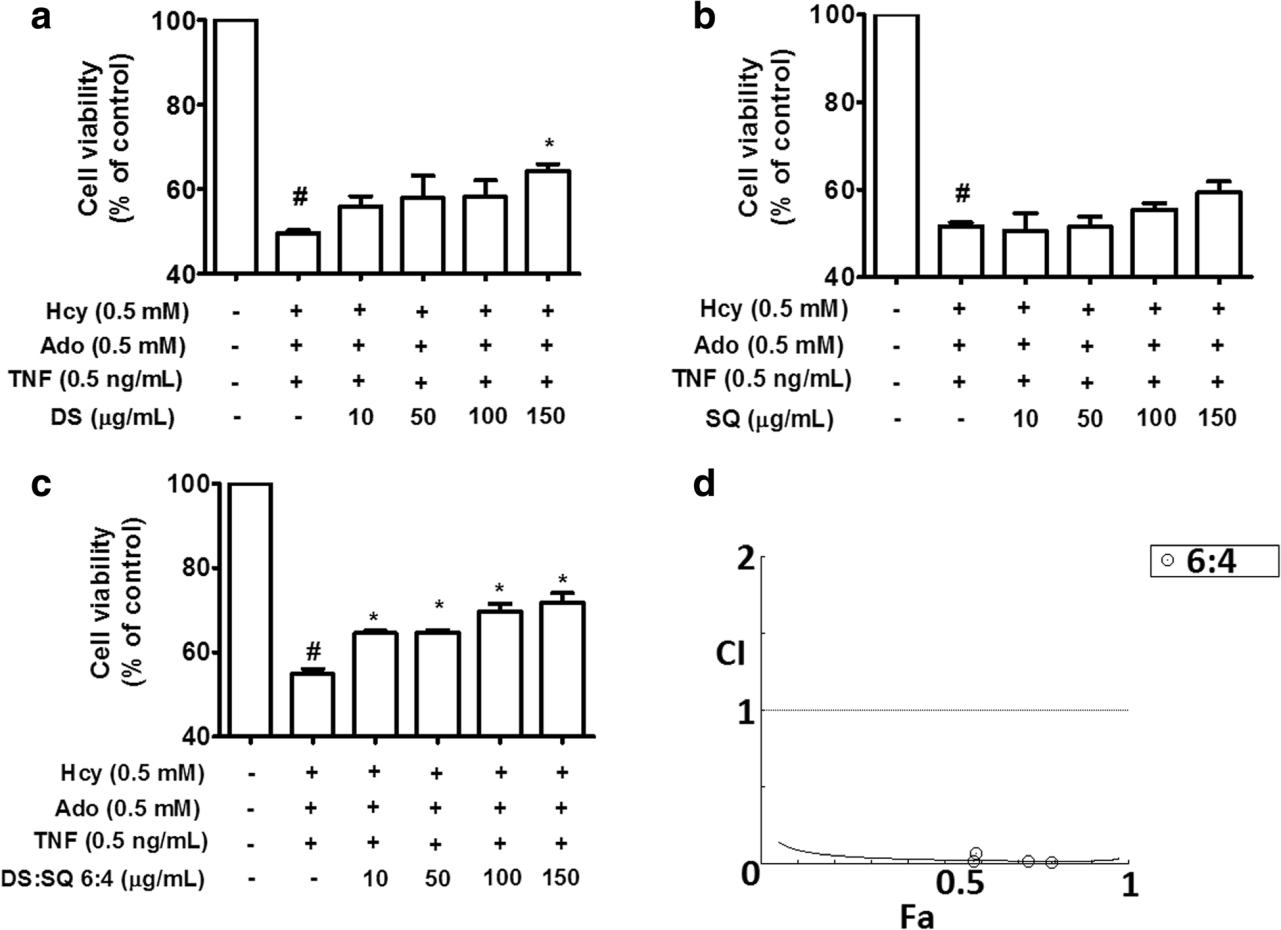
The protective effect of DS, SQ and DS-SQ (6:4) on EA.hy926 cells against Hcy-Ado-TNF cytotoxicity was determined by MTT assay. Cell viability, as determined for DS (**a**), SQ (**b**) and DS-SQ 6:4 (**c**) following treatments with Hcy-Ado-TNF in EA.hy926 cells, respectively. Cell viability was expressed as a percentage compared to control. Data shown are mean ± SEM, *n* > 3. * *P* < 0.05, ** *P* < 0.01, *** *P* < 0.001 in comparison with other combinations in the respective cell lines. **d** CI values were plotted as a function of fractional of cell viability (Fa) by ‘CompuSyn’ software. Fa values correspond to percentage cell viability relative to control. The solid line is the reference line, where CI value equals 1; solid line represents CI values at different Fa

As illustrated in Fig. 1, DS significantly restored cell viability at 150 μg/mL (*P* < 0.05), however, SQ extract (10–150 μg/mL) failed to reach statistical significance (*P* > 0.05). Except for DS to SQ ratio at 9:1, herbal-pair extracts at all tested ratios significantly restored impaired cell viability in a concentration-dependent manner from 10 to 150 μg/mL (*P* < 0.05). When the activity of combinational extracts at the same concentration level was compared, DS-SQ extracts at 6:4 ratios (10–150 μg/mL) showed the highest cell viability and this was constantly higher compared to DS or SQ alone. Figure 1d demonstrated the synergistic cytoprotective effects in DS-SQ 6:4 (10–150 μg/mL) via the MTT assay (CI < 1).

#### Synergistic effects of DS, SQ and DS-SQ 6:4 on LDH leakage suppression

**Fig. 2:**
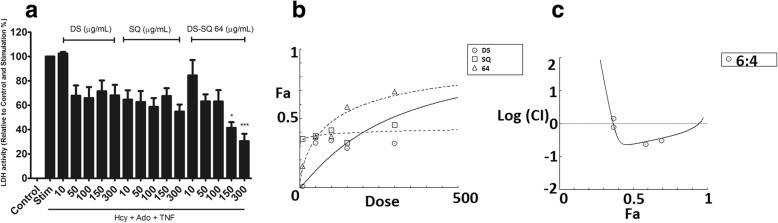
Inhibitory effects of DS, SQ and DS-SQ 6:4 on elevated levels of LDH leakage in EA.hy926 cells under cellular damage induced by Hcy-Ado-TNF. **a**. Inhibitory activity (% relative to control) of DS, SQ and DS-SQ 6:4 (10–300 μg/mL) on increased LDH activity (relative to control). Data shown are mean ± SEM, n > 3. **b**. Concentration-effect curves of LDH inhibitory activity of DS, SQ and DS-SQ 6:4 generated by CompuSyn software. **c**. Log (CI) values were plotted as a function of suppressing LDH leakage (Fa) by ‘CompuSyn’ software. Dotted line is the reference line, where Log (CI) value equals to 0; solid line represents CI values at different Fa. Fa values correspond to % LDH inhibitory effect relative to control

EA.hy926 cells incubated with Hcy-Ado-TNF for 4 h caused a significant increase in LDH leakage compared to the control group (*P* < 0.01), indicating significant cell damage. As Fig. 2a demonstrates, all single and combinational extracts (6:4) attenuated the increased LDH leakage in a concentration-dependent manner from 10 to 300 μg/mL. However, only DS-SQ 6:4 showed a statistically significant inhibitory effect on LDH at the concentrations of 150 and 300 μg/mL which were greater than DS and SQ alone at the same concentration levels (*P* < 0.05 and *P* < 0.001, respectively) (Fig. 2a). Synergistic effects were observed when the suppressive effect of DS-SQ 6:4 was greater than 38% (Fa > 0.38), as shown in Fig. 2c. The synergistic effect was stronger until the LDH inhibitory level reached 94%, and then it tended to be additive when the effect was predicted to be higher than 95%. The doses of DS and SQ in the combination were generally much lower than that of DS and SQ used alone to reach the same effect. For example, at 50% suppressive effect level, 69.32 and 46.22 μg/mL was needed for DS and SQ in the combination, respectively, which was much lower than that of DS (287.18 μg/mL) and SQ (35,407.2 μg/mL, predicted by CompuSyn) used alone to reach the same effect.

#### Synergistic effects of DS, SQ and DS-SQ 6:4 on caspase-3 total protein suppression

**Fig. 3:**
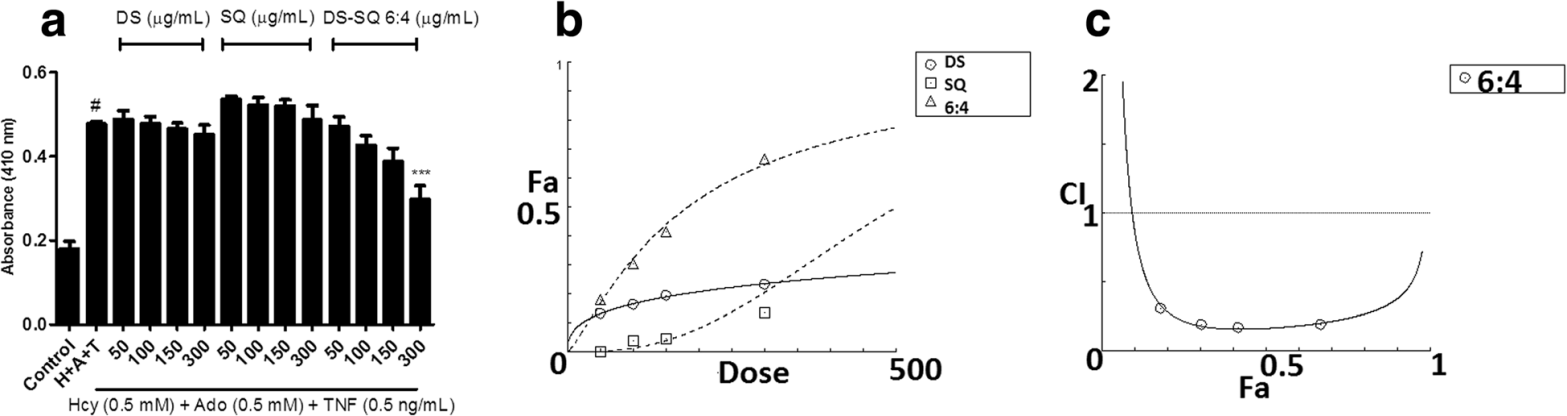
Inhibitory effects of DS, SQ and DS-SQ 6:4 on elevated levels of caspase-3 total protein expression in EA.hy926 cells under cellular damage induced by Hcy-Ado-TNF. **a**. The protein expression levels of caspase-3 were estimated by quantitative caspase colorimetric assay (R&D system, Australia). Results were shown as absorbance by microplate reader, with the means ± SEM, *n* > 3. * *P* < 0.05 vs. Hcy-Ado-TNF group. Absorbance (410 nm) versus concentrations (50–300 μg/mL) graph for DS, SQ and DS-SQ 6:4 in inhibiting increased caspase-3 protein expressions. ‘H + A + T’ represents Hcy-Ado-TNF stimulation only. Data shown are means ± SEM, n > 3. * *P* < 0.05 vs H + A + T group; *** *P* < 0.001 vs H + A + T group. **b**. Concentration-effect curves of caspase-3 inhibitory activities of DS, SQ and DS-SQ 6:4 generated by CompuSyn software. **c**. CI values were plotted as a function of suppression of caspase-3 proteins level (Fa) by CompuSyn software. The dotted line is the reference line, where CI value equals 1; solid line represents CI values at different Fa. Fa values correspond to % caspase-3 inhibitory effect relative to control

EA.hy926 cells incubated with Hcy-Ado-TNF for 4 h caused a significant increase in caspase-3 total protein expression compared to the control group (*P* < 0.05), indicating significant cell apoptosis. As shown in Fig. 3a, the single extract of DS and SQ from 50 to 300 μg/mL did not cause significant attenuation of the increased caspase-3 total protein levels, compared to the Hcy-Ado-TNF group (*P* > 0.05). However, a significant inhibitory effect was observed with DS-SQ 6:4 at 300 μg/mL (*P* < 0.001). A synergistic effect of DS-SQ 6:4 was generally observed in inhibiting caspase-3 total protein production (Fig. 3c). The synergistic effect already became apparent at 10% effect level (Fa = 0.1) and continued to increase at higher effect levels.

#### Synergistic effects of bioactive compounds on impaired cell viability

**Fig. 4:**
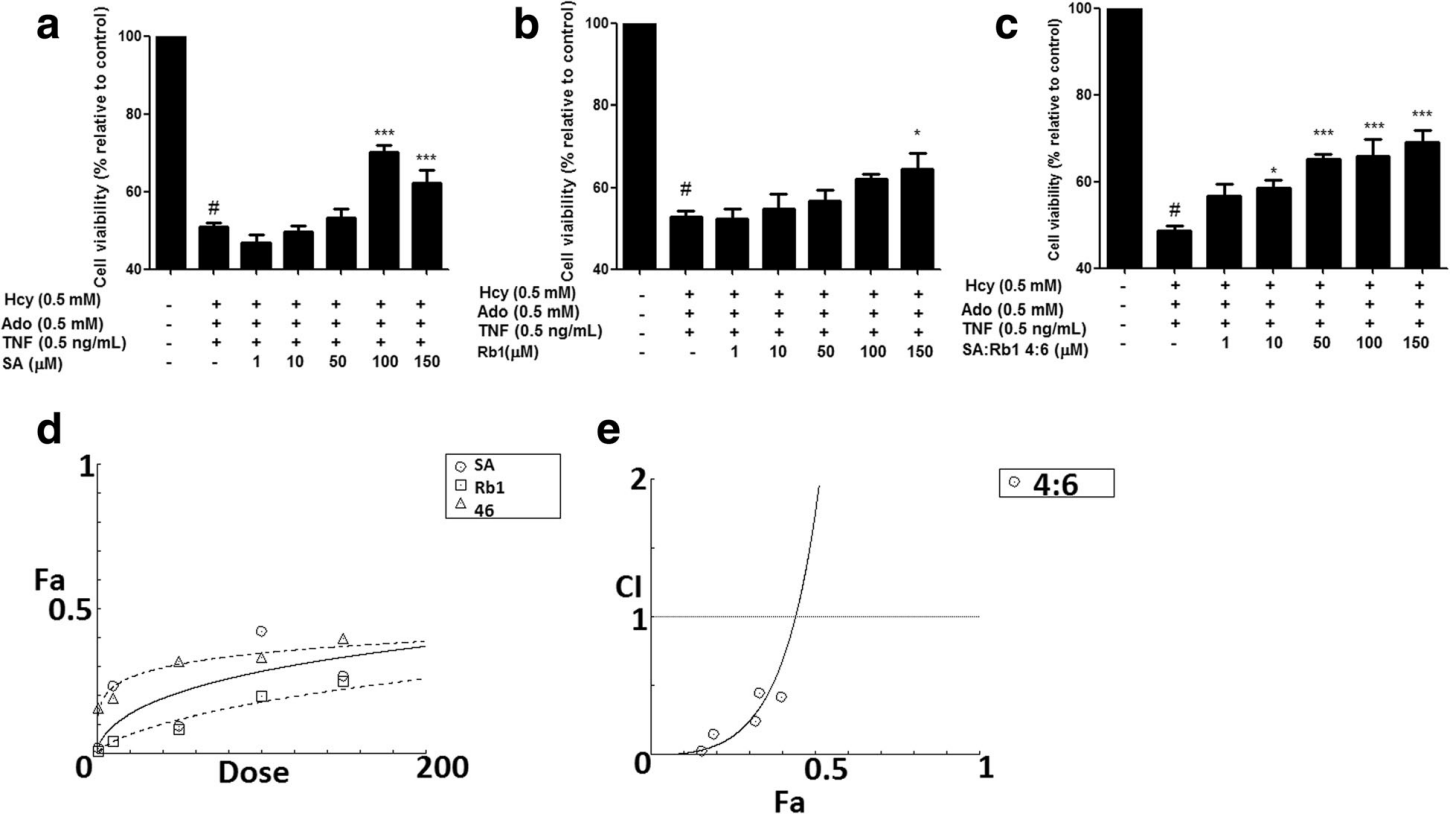
The protective effect of SA, Rb1 and SA-Rb1 4:6 on EA.hy926 cells against cytotoxicity induced by Hcy-Ado-TNF was determined by MTT assay. Cell viability, as determined by MTT for SA (**a**), Rb1 (**b**) and SA-Rb1 4:6 (**c**) following treatments with Hcy-Ado-TNF in EA.hy926 cells. Cell viability was expressed as a percentage compared to control. Data shown are mean ± SEM, n > 3. * *P* < 0.05, ** *P* < 0.01, *** *P* < 0.001 in comparison to other combinations in the respective cell lines. **d** Concentration-effect curves of SA, Rb1 and SA-Rb1 4:6 (10–150 μM) on restored cell viability generated by CompuSyn software. **e** CI values were plotted as a function of fractional restored cell viability (Fa) by CompuSyn software. Fa values correspond to % cell viability relative to control. Dotted line is the reference line, where CI value equals 1; solid line represents CI values at different Fa

Fourteen bioactive compounds from DS and SQ were tested for their cytoprotective effects against the cellular damage induced by Hcy-Ado-TNF, using MTT assay. Among all compounds from DS (Additional file 4), only SA showed improvement in cell viability in a dose-dependent manner. As shown in Fig. 4, SA significantly restored the cell viability at the concentration greater than 100 μM (*P* < 0.05). SB, the most abundant bioactive compound in DS, did not show any significant protective effects (*P* > 0.05). Among all the tested compounds in SQ (Additional file 5), only ginsenoside Rb1 showed significant improvement at 150 μM (P < 0.05). Therefore, SA and Rb1 from DS and SQ, respectively, were found to be the most active compounds in protecting EA.hy926 cells from Hcy-Ado-TNF. Thus, SA and Rb1 were further studied by combining the specified ratios (1:9, 2:8…8:2, 9:1) and tested using MTT assays. The results suggested that SA-Rb1 4:6 demonstrated significant effects when the tested concentration was greater than 10 μM (*P* < 0.05, Fig. 4). Synergistic effects were generally observed for SA-Rb1 4:6 ratio at all tested concentrations (1–150 μM) for restoring impaired cell viability on MTT assay (CI < 1, Fig. 4e). The synergistic interaction tended to be weaker when the concentration of SA-Rb1 was higher than 150 μM. The interaction was predicted to be antagonistic, when the concentration was higher than 465.51 μM.

#### Synergistic effects of SA-Rb1 on LDH leakage suppression

**Fig. 5:**
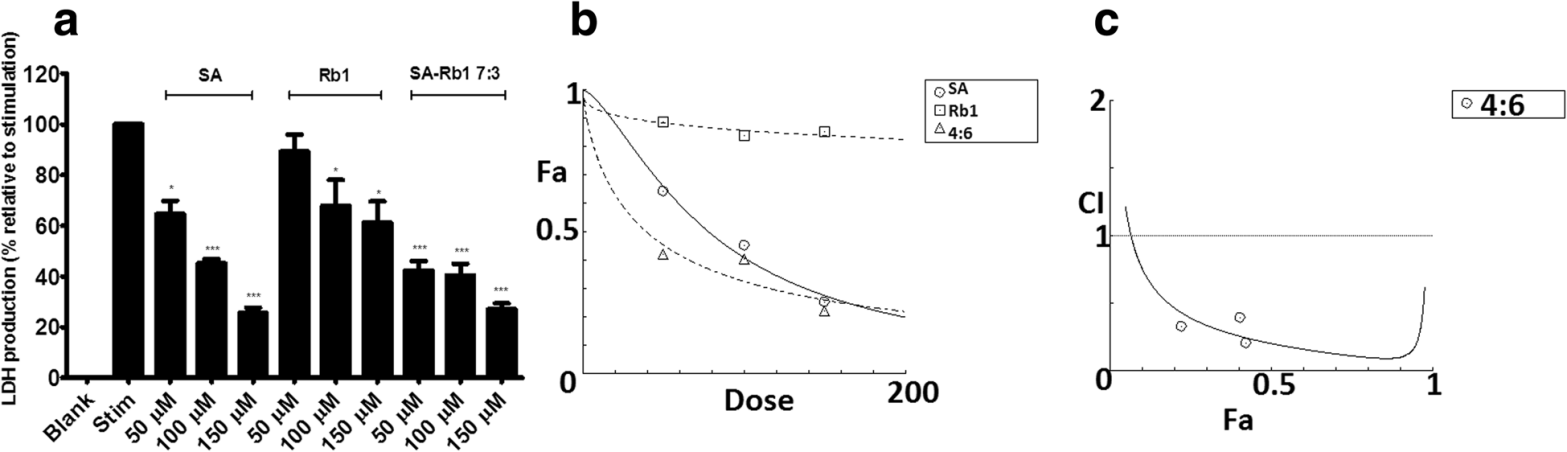
Effects of SA, Rb1 and SA-Rb1 4:6 in inhibiting LDH leakage on EA.hy 926 cells against cellular damage induced by Hcy-Ado-TNF. **a** Inhibitory activity (% relative to control) of SA, Rb1 and SA-Rb1 4:6 (50–150 μM) in inhibiting increased LDH activity (relative to control). Data shown are means ± SEM, n > 3. * *P* < 0.05 vs. stim group; *** *P* < 0.001 vs. stim group; ‘stim’ represents Hcy-Ado-TNF stimulation. **b** Concentration-effect curves of LDH inhibitory effects of SA, Rb1 and SA-Rb1 4:6 generated by CompuSyn software. **c** CI values were plotted as a function of suppression fractional of LDH leakage (Fa) by ‘CompuSyn’ software. Fa values correspond to % LDH inhibitory effect relative to control. Dotted line is the reference line, where CI value equals 1; solid line represents CI values at different Fa

As shown in Fig. 5a, SA, Rb1 and SA-Rb1 (4:6) significantly reduced the LDH leakage induced by Hcy-Ado-TNF at all tested concentrations (P < 0.05), except for Rb1 at 50 μM (*P* > 0.05). A strong synergistic effect (CI < 1 with Fa from 0.1 to 0.97) was generally shown at the tested concentration range (50–150 μM) for SA-Rb1 4:6 (Fig. 5c). The doses of SA and Rb1 in the combination were generally much lower than that of SA and Rb1 used alone to reach the same effect. For example, to reach 50% suppressive level, 15.82 and 23.73 μM were needed for SA and Rb1 in the combination, respectively, and the doses were much lower than that of SA (78.1074 μM) and Rb1 (20,697 μM, predicted by CompuSyn) used alone to reach the same effect.

### Synergistic effects of DS, SQ, DS-SQ 6:4 and their bioactive compounds on H_2_O_2_ impaired cell survival in EA.hy926 cells

#### Synergistic effects of DS, SQ and DS-SQ on impaired cell viability

**Fig. 6:**
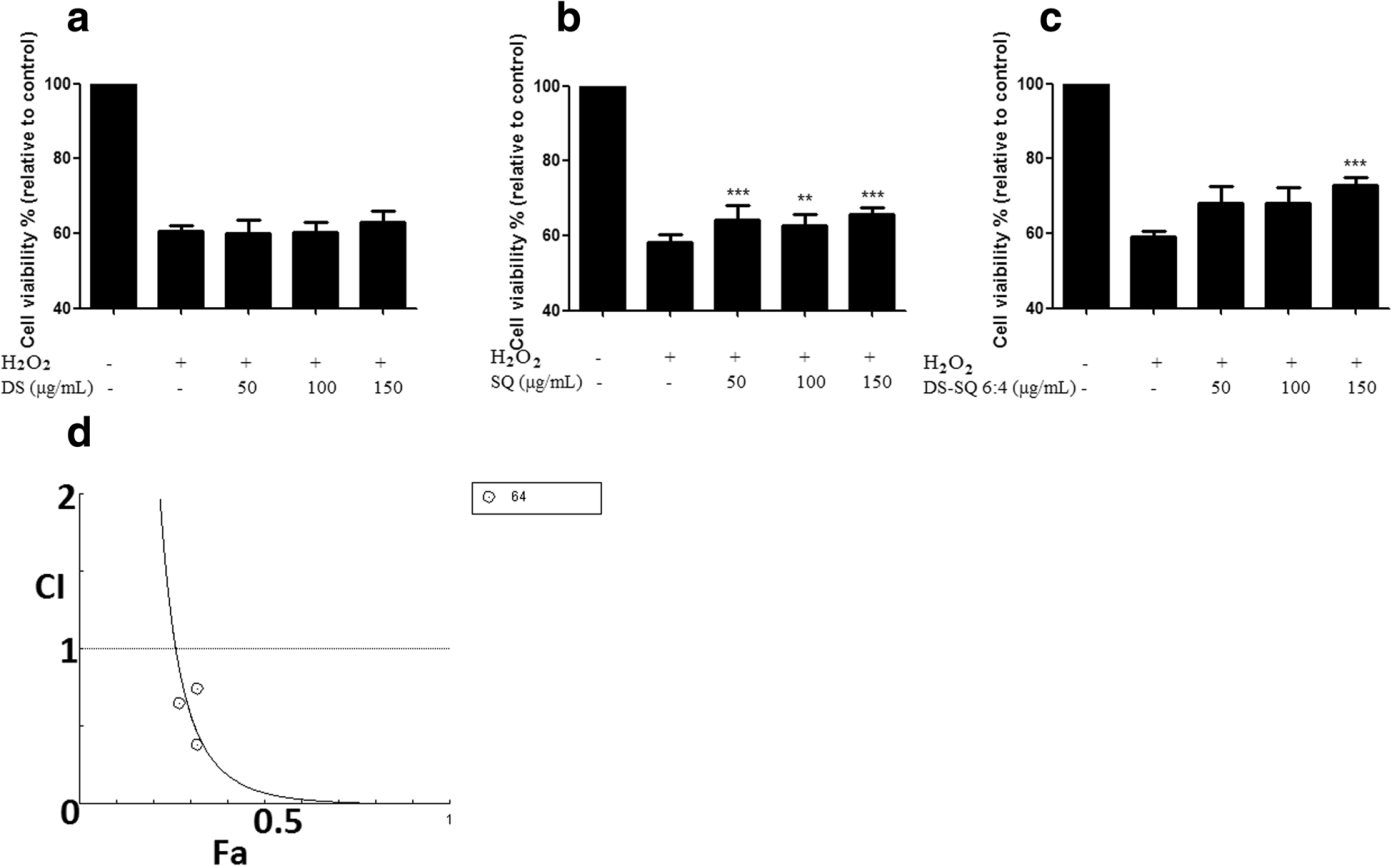
Protective effect of DS, SQ and DS-SQ 6:4 on EA.hy926 cells against H_2_O_2_-induced cell injury by restoring reduced cell viability. Cell viability was assayed by the MTT method (*n* = 4) in EA.hy926 cells for 20 h. Cell viability was expressed as a percentage compared to H_2_O_2_ stimulation only. Data shown are means ± SEM, *n* > 3. Cell viability (% relative to control) versus increased concentrations of (**a**) DS, (**b**) SQ and (**c**) DS-SQ 6:4 (10–300 μg/mL) against H_2_O_2_ induced cell damage. #*P* < 0.05 vs control. * *P* < 0.05 vs H_2_O_2_ treatment only. ** *P* < 0.01 vs H_2_O_2_ treatment only. *** *P* < 0.001 vs H_2_O_2_ treatment only. **d**. CI values were plotted as a function of restored cell viability (Fa) by ‘CompuSyn’ software. Fa values correspond to restored cell viability % relative to control. Dotted line is the reference line, where CI value equals 1; solid line represents CI values at different Fa

H_2_O_2_ (0.1–10 mM) concentration-dependently reduced cell viability. As 0.5 mM H_2_O_2_ caused a significant cellular injury (*P* < 0.001), this concentration was selected for the H_2_O_2_ induced cell injury model. The cell viability with pre-treatment of DS, SQ and DS-SQ 6:4, followed by H_2_O_2_ (0.5 mM), was shown in Fig. 6. There were no significant protective effects from DS on restoring impaired cell viability against H_2_O_2_ (*P* > 0.05). SQ (50–150 μg/mL) showed concentration-dependent effects and significantly restored the impaired cell viability induced by H_2_O_2_ (*P* < 0.05). It was found that DS-SQ 6:4 (150 μg/mL) significantly restored cell viability against H_2_O_2_ (0.5 mM) (*P* < 0.01). Synergistic effect was generally observed for DS-SQ 6:4 in restoring impaired cell viability after the cells were exposed to H_2_O_2_, with CI values of 0.072 at ED_50_ (Fig. 6d).

#### Synergistic effects of DS, SQ and DS-SQ on ROS generation

**Fig. 7:**
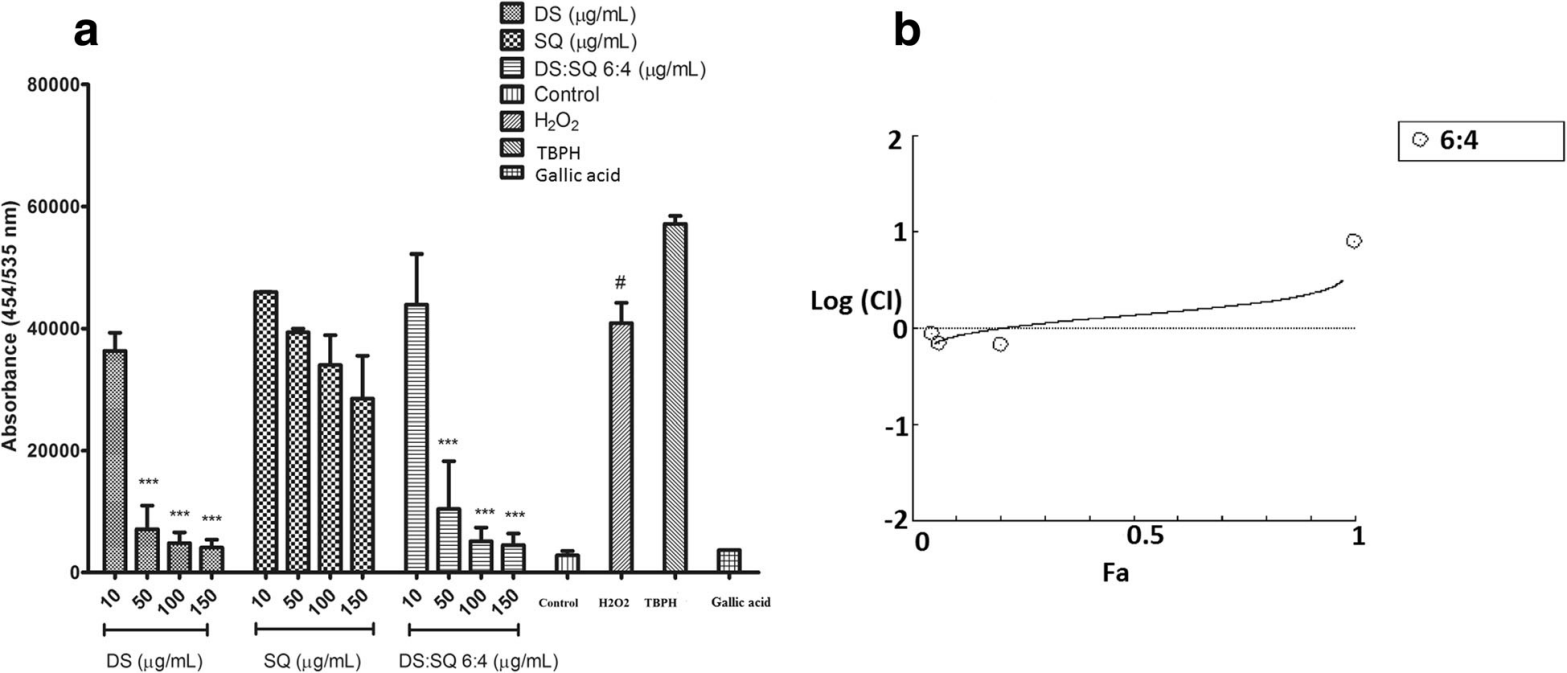
Protective effects of DS, SQ and DS-SQ 6:4 against H_2_O_2_-induced endothelial cell injury by attenuating excessively expressed ROS production. **a** Inhibitory effect of DS, SQ and DS-SQ 6:4 on ROS production induced by H_2_O_2_ in EA.hy926 cells. The ROS expression level was estimated by cellular ROS detection assay kit manufacturer (Abcam, Australia). TBPH (from kit) was used as negative control and gallic acid (10 μg/mL) was used as a positive control in the assay. Results were shown as absorbance by a microplate reader, with the means ± SEM, *n* > 3. *** *P* < 0.001 compared with H_2_O_2_ group. **b** Log (CI) values were plotted as a function of suppression fractional of ROS level (Fa) by ‘CompuSyn’ software. Dotted line is the reference line, where Log (CI) value equals to 0; solid line represents Log (CI) values at different Fa. Fa values correspond to percentage ROS inhibitory effect relative to control

As shown in Fig. 7, EA.hy926 cells incubated with H_2_O_2_ resulted in a significant increase of ROS production compared to the control group (*P* < 0.01). Pre-treatment with DS (10–150 μg/mL) concentration-dependently attenuated the ROS generation by H_2_O_2_, with significance at 50–150 μg/mL. SQ extract showed no significant effect in inhibiting ROS from 10 to 150 μg/mL. DS-SQ 6:4 showed significant activity in decreasing the ROS generation at 50, 100 and 150 μg/mL compared to the H_2_O_2_ group (*P* < 0.001). Gallic acid was used as a positive control in this assay, as its prominent anti-oxidant activity showed significant ROS inhibitory effect at 10 μg/mL. A one-way ANOVA test suggested that the inhibitory effect of DS and DS-SQ 6:4 at 150 μg/mL were comparable with the effect of gallic acid (10 μg/mL) (*P* > 0.05). A synergistic effect was also observed for DS-SQ 6:4 at lower effects in inhibiting ROS production induced by H_2_O_2_ (Figs. 7b), when the Fa level (ROS inhibitory effect) was lower than 0.2 (20% inhibition), and the concentration range for DS-SQ 6:4 was calculated to be smaller than 80.51 μg/mL. Whereas, the antagonistic effect was observed when the concentration of DS-SQ 6:4 was above 80.511 μg/mL. The CI values were calculated to be 1.39 and 2.31 at ED_50_ and ED_90_, respectively.

#### Synergistic effect of SA-Rb1 4:6 on impaired cell viability

**Fig. 8:**
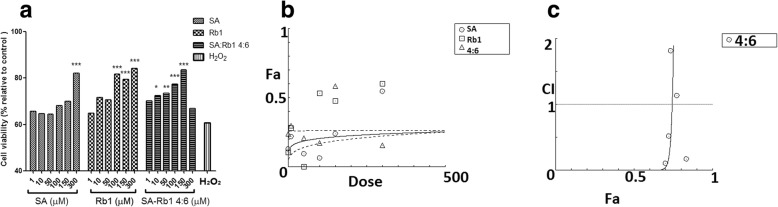
Protective effect of SA, Rb1 and SA-Rb1 4:6 on EA.hy926 cells against H_2_O_2_-induced cell injury was determined by MTT assay. **a** Cell viability (% relative to control) verses increased concentrations of SA, Rb1 and SA-Rb1 4:6 (1–150 μM) against H_2_O_2_ induced cell damage. **b** Concentration-effect curves of restored cell viability against H_2_O_2_ treatment with increasing concentrations of SA, Rb1 and SA-Rb1 4:6 (1–150 μM) were generated by CompuSyn software. Data shown are mean ± SEM, *n* > 3. **c** CI values were plotted as a function of restored cell viability (Fa) by ‘CompuSyn’ software. Fa. Fa values correspond to restored cell viability % relative to control. Dotted line is the reference line, where CI value equals 1; solid line represents CI values at different Fa

The cell viability with pre-incubation of SA, Rb1, and SA-Rb1 (4:6) with different concentrations (1–300 μM) followed by H_2_O_2_ (0.5 mM) cell injury was shown in Fig. 8. Both SA (300 μM) and Rb1 (100–300 μM) showed significant effects for restoring the impaired cell viability of H_2_O_2_-treated EA.hy926 cells (*P* < 0.05). Additionally, a prominent effect was observed for SA-Rb1 4:6 (1–150 μM), with significant improvement of cell viability when the concentration was greater than 10 μM (*P* < 0.001). Similarly, a synergistic effect for SA-Rb1 4:6 was observed against H_2_O_2_-induced cell damage with concentration less than 35.14 μM. At the above concentrations, it became antagonistic as the protective effect of SA alone was stronger than the combination (Fig. 8c).

#### Synergistic effect of SA-Rb1 4:6 on ROS production

**Fig. 9:**
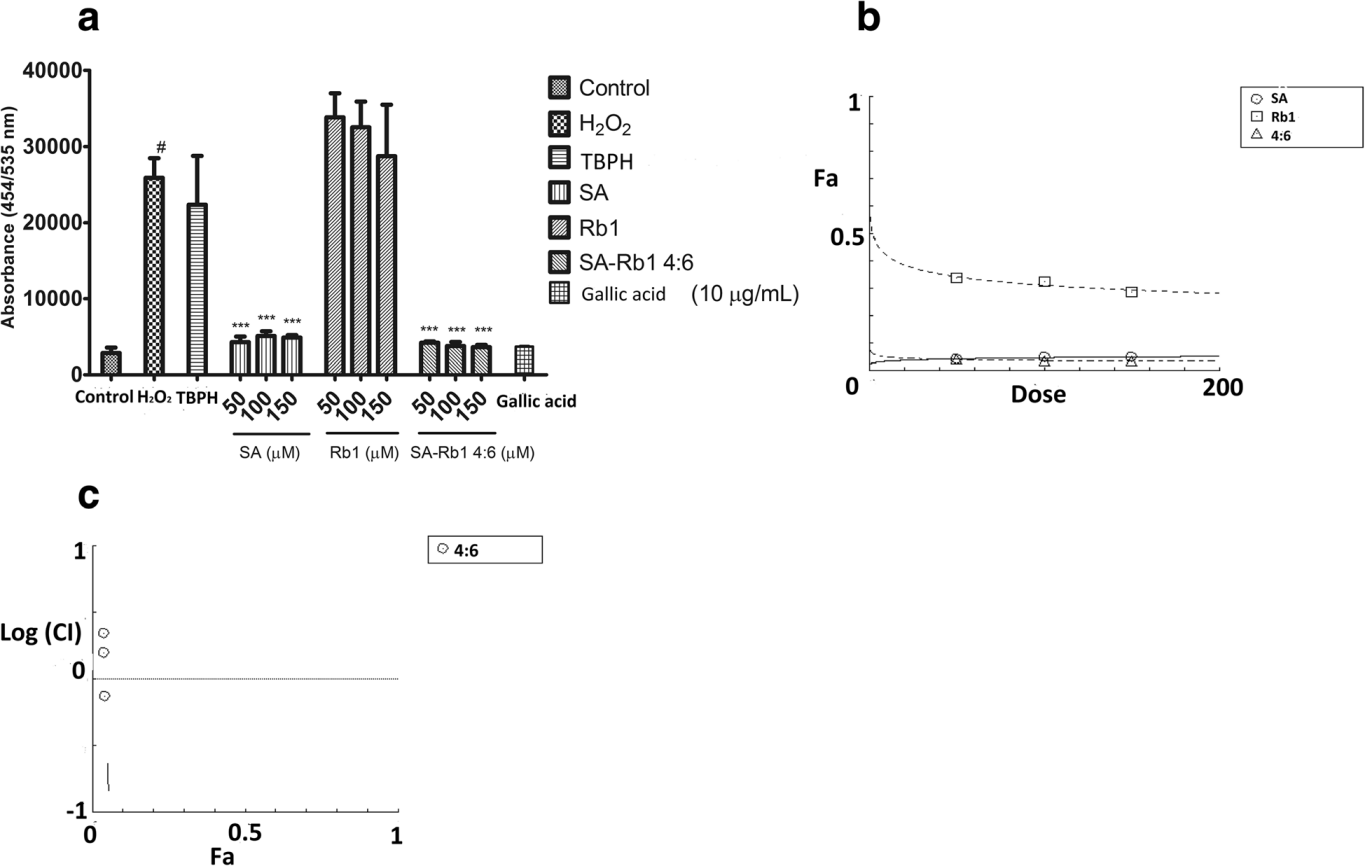
Protective effect of SA, Rb1 and SA-Rb1 4:6 on EA.hy926 cells against H_2_O_2_-induced cell injury by inhibiting excessive intracellular ROS production. **a** Inhibitory effect of SA, Rb1 and SA-Rb1 4:6 on ROS production induced by H_2_O_2_ in EA.hy926 cells. The ROS expression level was estimated by cellular ROS detection assay kit manufacturer (Abcam, Australia). TBPH (from kit) was used as positive control in the assay. Results were shown as absorbance by a microplate reader, with the means ± SEM, n > 3. **b** Concentration-effect curves of ROS inhibitory effects of SA, Rb1 and SA-Rb1 4:6 generated by CompuSyn software. **c** Log (CI) values were plotted as a function of suppression fractional of ROS (Fa) by ‘CompuSyn’ software. Fa values correspond to % ROS inhibitory effect relative to control. Dotted line is the reference line, where Log (CI) value equals 0; solid line represents CI values at different Fa

As shown in Fig. 9, both SA and SA-Rb1 significantly inhibited ROS production from 50 to 150 μM (*P* < 0.001), whereas Rb1 did not show any significant inhibitory effect for ROS production (*P* > 0.05). Additionally, gallic acid (10 μg/mL) was used as a negative control in this assay, which inhibited ROS significantly. The non-parametric test suggested that the inhibitory effect of SA and SA-Rb1 4:6 from 50 to 150 μM was comparable to that of gallic acid (P > 0.05). For the inhibitory effect of SA-Rb1 4:6 on ROS production, a synergistic effect was only observed below 50 μM. It was predicated to be antagonistic at concentrations greater than 100 μM (Fig. 9c).

## Discussions

This study shows for the first time the cytoprotective activity of SQ extract and compounds of both DS and SQ in endothelial cells. Further to previous studies, we applied CI model and systematically analysed the possible synergistic effects for the DS-SQ combinations with nine ratios (1:9 to 9:1, *w*/w) in protecting endothelial cell injury against both Hcy-Ado-TNF and H_2_O_2_. Our results revealed that the DS-SQ paired herb at all tested ratios restored impaired cell injury against Hcy-Ado-TNF. The combinational effect was more prominent than the single herbs at the same concentration level. This suggested the superior effects of using the combination for enhanced bioactivity on the same target.

Among all the tested combinations, DS-SQ 6:4 was found to be the optimal ratio for its potent protective effects compared to that of other ratios, not only for restoring cell viability, but also reducing the cell apoptosis (LDH, caspase-3). Furthermore, this particular combination consistently restored the cell injury against oxidative stress by directly suppressing ROS level. For the first time, this finding suggests that synergistic interactions exist with the DS and SQ combination in protecting endothelial cells against both Hcy-Ado-TNF- and oxidative stress-mediated cell injury.

Moreover, the observed synergistic effect is concentration-dependent as revealed by CI. These results suggest that the interactions in the combination are dependent on the composition of the ingredients. In TCM clinical practice, the ratio (dosage) of herbal ingredients is tailored to individual patient in order to reach a desirable clinical outcome based on syndrome differentiation treatment strategy. Our findings provide the preliminary evidence for this theory. The optimal ratio of DS and SQ against cytotoxicity on EA.hy926 cells tended to be equal in this study. In traditional practice, a higher ratio (dosage) of DS in the combination is usually used for conditions at the early stage of disease, when pathological changes in the organs are not obvious (possibly pointing to the early inflammatory stage), and the proportion of SQ should be increased at the later stage of the disease, when pathological changes have been diagnosed (later stage for vascular dysfunction) [42]. Our observations support this theory and clinical use, however further in vivo and human studies are needed to confirm this finding. There are several possible mechanisms for the pharmacodynamics synergy in combinational therapy, including increased extraction yield of bioactive compounds, chemical reactions among compounds, multi-target behaviour or enhanced bioavailability [43]. While contradictory, current chemical analysis studies on the  DS-SQ combination indicate that the chemical composition of DS-SQ mixed preparation may not simply be the sum of chemical constituents from the single herb, and the new compounds are likely formed during the mixture preparation which contribute to the enhanced bioactivity of the combination. Our ultra-performance liquid chromatography chemical analysis for the combination revealed that the chemical fingerprint was altered in the combined extract comparing to that of individual extract, and the amount of bioactive compounds (DSS, SB, NR1, Rg1 and Rb1) was not directly proportional to the ratio of their herbal ingredients in the combination (Additional file 2).

To investigate the mechanistic actions of the observed synergistic actions for the mixed extracts by multi-target behaviour, the effects of fourteen key bioactive compounds of DS and SQ were further investigated using the same assays. SA and Rb1 were found to be effective in restoring impaired cell injury against Hcy-Ado-TNF and oxidative stress. Other tested compounds showed no significant effects in the current experimental condition, indicating that these compounds may be inactive on the cellular mechanisms studied. However, this also does not exclude the possibility that these compounds may become active in vivo due to metabolism or transformation [44]. Additionally, the present findings do not exclude the possibility of the involvement of the other active components in the actions of the DS-SQ extract, as more than 100 single compounds have currently been isolated from DS and SQ. A systems biology approach can be applied to predict the key constituents for the potential targets, and the experimental data can be confirmed by bioassay-guided fractionation. Previously, the multi-target behaviour from DS-SQ was demonstrated in several studies using systems biology. For example, Li et al. (2012) established a systems biology model for DS-SQ on cardiovascular diseases targets and found that the candidate compounds from DS tackled 39 out of the total 41 validated CVD targets, whereas those from SQ interacted with 36 potential targets, 34 of which overlapped with DS’s targets [45].

In addition, synergistic effects were generally observed for SA-Rb1 combination on anti-apoptosis. This demonstrated that the combination of two prominent compounds may contribute to the effects of the mixed extract and engender a stronger effect. The two compounds may act as an agonist for each other on the same receptor, thus the effect is enhanced. This simplified compounds combination which maintained the effect and exhibited synergy maybe a potential pharmaceutical agent to be used on endothelial dysfunction. However, the effect and toxicity need to be further confirmed in in vivo and human studies.

## Conclusions

DS-SQ 6:4 showed prominent and synergistic effects in restoring reduced cell viability and attenuating excessively expressed LDH and caspase − 3 against Hcy-Ado-TNF. In addition, this combination exerted a synergistically-enhanced cytoprotective effect from oxidative damage (caused by H_2_O_2_) by restoring the cell viability and inhibiting excessive ROS production. SA and Rb1 were found to be the most potent component in each extract for restoring reduced cell viability. Furthermore, SA-Rb1 4:6 combination showed synergistically -enhanced effects in preventing cytotoxic effects caused by Hcy-Ado-TNF as analysed by CI. This simplified combination demonstrated prominent effects on H_2_O_2_-induced oxidative damage. Our findings provided a theoretical basis for the purported synergistic efficiency of DS and SQ as a herbal drug pair on vascular function through its protection on endothelial cells against major stressors: Hcy, TNF and oxidative stress.

## Additional files

Additional file 1: Chemical fingerprint of raw herb extract of DS and SQ analysed by UPLC-PDA. (PDF 110 kb)

Additional file 2: Contents (mg/g, mean ± SD, *n* = 3) of DSS, SB, NR1, Rg1 and Rb1 in DS-SQ combinations extract by UPC-PDA. (PDF 43 kb)

Additional file 3: Cytotoxic effects of Hcy-Ado-TNF on EA.hy926 cells as determined by MTT dye reduction assay, following treatments with Hcy-Ado-TNF in EA.hy926 endothelial cells for 20 h. (DOCX 27 kb)

Additional file 4: Effects of bioactive compounds from DS on restoring cell viability against Hcy-Ado-TNF in EA.hy926 cells determined by MTT assay. (DOCX 284 kb)

Additional file 5: Effects of bioactive compounds from SQ on restoring cell viability against Hcy-Ado-TNF in EA.hy926 cells determined by MTT assay. (DOCX 282 kb)

## Abbreviations

**Ado**: Adenosine
BMSCs: Bone marrow stromal cells
CI: Combination index
**CIR**: Cerebral ischaemia reperfusion
CT: Cryptotanshinone
CT: Cryptotanshinone
**DCFDA**: 2′,7′ –dichlorofluorescin diacetate
DMEM: Dulbecco’s Modified Eagle’s Medium
DMSO: Dimethyl sulfoxide
DSS: Sodium danshensu
DS-SQ: Danshen-Sanqi extracts
DT: Dihydrotanshinone I
EA.hy926: Human cardiovascular endothelial
eNOS: Endothelial nitric oxide synthase
Hcy: Homocysteine
HUVECs: Human umbilical vein endothelial cells
LDH: Lactate dehydrogenase
MAPK: Mitogen-activated protein kinase
NR1: Notoginsenoside R1
PDGF: Platelet-derived growth factor
**Penstrep**: Penicillin and streptomycin
R**b1**: Ginsenoside Rb1
R**b1**: Ginsenoside Rb1
Rd.: Ginsenoside Rd.
**Rg1**: Ginsenoside Rg1
**Rg2**: Ginsenoside Rg2
ROS: Reactive oxygen species
ROS: Reactive oxygen species
SA: Salvianolic acid A
SB: Salvianolic acid B
TCM: Traditional Chinese Medicine
TI: Tanshinone I
TIIA: Tanshinone IIA
TNF: Tumour necrosis factor
