# Supplementary material for: Synergistic study of a Danshen (Salvia Miltiorrhizae Radix et Rhizoma) and Sanqi (Notoginseng Radix et Rhizoma) combination on cell survival in EA.hy926 cells
Source: BMC Complement Altern Med. 2019 Feb 21;19:50. doi: 10.1186/s12906-019-2458-z (PMC6385400; doi:10.1186/s12906-019-2458-z)
Supplement: Supplementary file 1 — Chemical fingerprint of raw herb extract of DS and SQ analysed by UPLC-PDA. (PDF 110 kb) [file 12906_2019_2458_MOESM1_ESM.pdf]

Additional file 1: Chemical fingerprint of raw herb extract of DS (A) and SQ (B) analysed by UPLC-PDA. (1) NR1, (2) Rg1, (3) Rb1, (4) Rg2, (5) Rd, (6) DSS, (7) SB, (8) SA, (9) DT, (10) CT, (11) T1, and (12) TIIA [1].

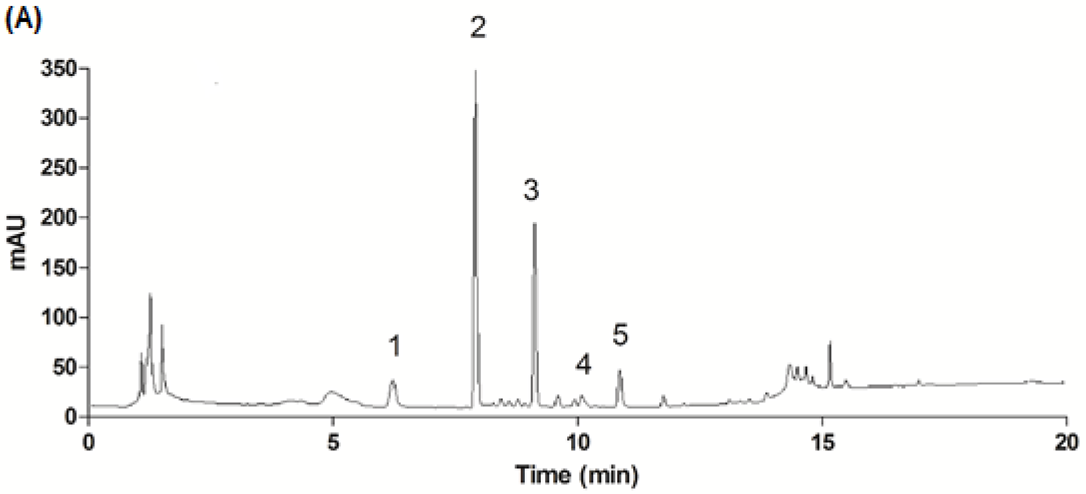

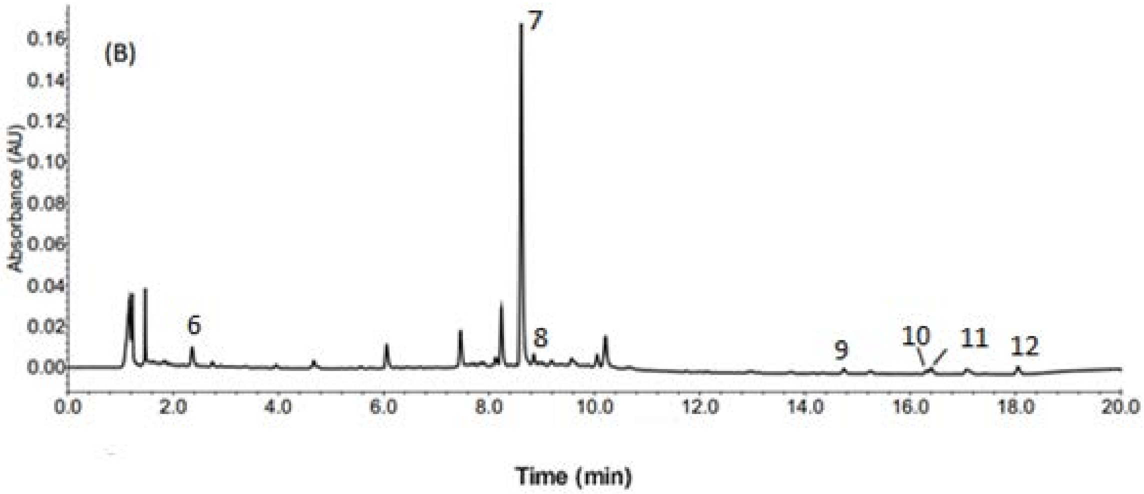

1. Zhou, X.; Razmovski-Naumovski, V.; Chan, K. A multivariate analysis on the comparison of raw notoginseng (Sanqi) and its granule products by thin-layer chromatography and ultra-performance liquid chromatography. *Chinese medicine* **2015,** *10,* (1), 13, doi: 10.1186/s13020-015-0040-2.
