# Supplementary material for: Synergistic study of a Danshen (Salvia Miltiorrhizae Radix et Rhizoma) and Sanqi (Notoginseng Radix et Rhizoma) combination on cell survival in EA.hy926 cells
Source: BMC Complement Altern Med. 2019 Feb 21;19:50. doi: 10.1186/s12906-019-2458-z (PMC6385400; doi:10.1186/s12906-019-2458-z)

Additional file 2: Contents (mg/g, mean±SD, n=3) of DSS, SB, NR1, Rg1, and Rb1 in DS-SQ combinations extract by UPLC-PDA.

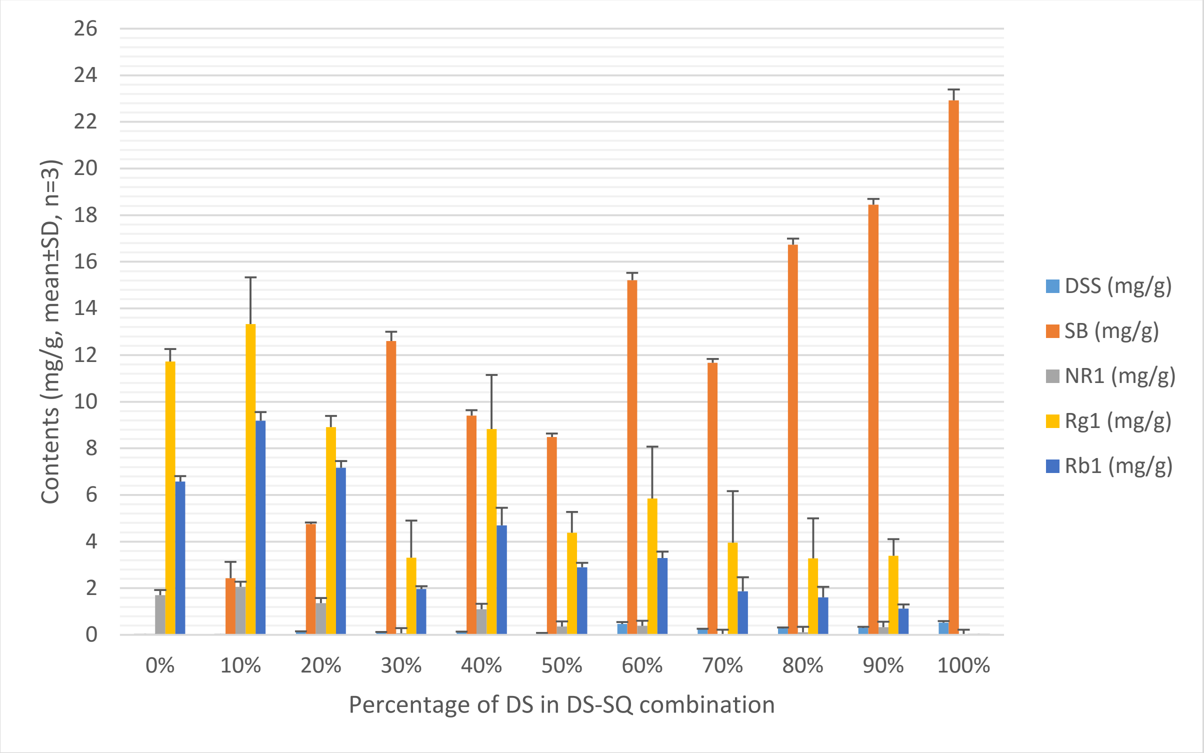

Supplement: Supplementary file 2 — Contents (mg/g, mean ± SD, n = 3) of DSS, SB, NR1, Rg1 and Rb1 in DS-SQ combinations extract by UPC-PDA. (PDF 43 kb) [file 12906_2019_2458_MOESM2_ESM.pdf]
