# Supplementary material for: Synergistic study of a Danshen (Salvia Miltiorrhizae Radix et Rhizoma) and Sanqi (Notoginseng Radix et Rhizoma) combination on cell survival in EA.hy926 cells
Source: BMC Complement Altern Med. 2019 Feb 21;19:50. doi: 10.1186/s12906-019-2458-z (PMC6385400; doi:10.1186/s12906-019-2458-z)

Additional file 3: Cytotoxic effects of Hcy-Ado-TNF on EA.hy926 cells as determined by MTT dye reduction assay, following treatments with Hcy-Ado-TNF in EA.hy926 endothelial cells for 20 h. Cell viability was expressed as a percentage compared to blank control (no Hcy, Ado or TNF). All results were expressed as mean ± S.E.M. from three separate experiments.


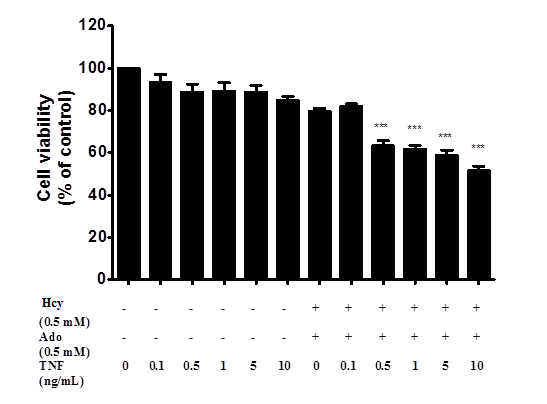

Supplement: Supplementary file 3 — Cytotoxic effects of Hcy-Ado-TNF on EA.hy926 cells as determined by MTT dye reduction assay, following treatments with Hcy-Ado-TNF in EA.hy926 endothelial cells for 20 h. (DOCX 27 kb) [file 12906_2019_2458_MOESM3_ESM.docx]
