# Supplementary material for: Synergistic study of a Danshen (Salvia Miltiorrhizae Radix et Rhizoma) and Sanqi (Notoginseng Radix et Rhizoma) combination on cell survival in EA.hy926 cells
Source: BMC Complement Altern Med. 2019 Feb 21;19:50. doi: 10.1186/s12906-019-2458-z (PMC6385400; doi:10.1186/s12906-019-2458-z)
Supplement: Supplementary file 5 — Effects of bioactive compounds from SQ on restoring cell viability against Hcy-Ado-TNF in EA.hy926 cells determined by MTT assay. (DOCX 282 kb) [file 12906_2019_2458_MOESM5_ESM.docx]

Additional 5: Effects of bioactive compounds from SQ on restoring cell viability against Hcy-Ado-TNF in EA.hy926 cells determined by MTT assay.

Effects of bioactive compounds from SQ on restoring cell viability against Hcy-Ado-TNF in EAhy 926 cells determined by MTT assay. Cell viability, as determined by MTT for NR1 (A), Rg1 (B), Re (C), Rb1 (D), Rb2 (E), Rg2 (F) and Rd (G) following treatments with Hcy-Ado-TNF in EAhy 926 cells. Cell viability was expressed as a percentage compared to control. ‘Stim’ here represents Hcy-Ado-TNF stimulation group. All results were expressed as mean ± S.E.M. from three separate experiments in triplicate. * P< 0.05, ** P<0.01, *** P < 0.001 in comparison with other combinations in the respective cell lines.
